# Supplementary material for: Magnetic control of self-assembly and disassembly in organic materials
Source: Nat Commun. 2023 May 29;14:3081. doi: 10.1038/s41467-023-38846-2 (PMC10227084; doi:10.1038/s41467-023-38846-2)
Supplement: Supplementary file 1 — Supplementary Information [file 41467_2023_38846_MOESM1_ESM.pdf]

SUPPLEMENTARY INFORMATION

# Magnetic control of self-assembly and disassembly in organic materials

*You-jin Jung*[1,†]*, Hyoseok Kim*[1,†]*, Hae-Kap Cheong*[2]*, and Yong-beom Lim*[1]*

[1]Department of Materials Science & Engineering, Yonsei University, 50 Yonsei-ro, Seoul 03722, Republic of Korea

[2]Division of Magnetic Resonance, Korea Basic Science Institute, Ochang 28119, Republic of Korea

[†]These authors contributed equally: You-jin Jung, Hyoseok Kim

*E-mail: yblim@yonsei.ac.kr

**a**

***Rod-coil building blocks***

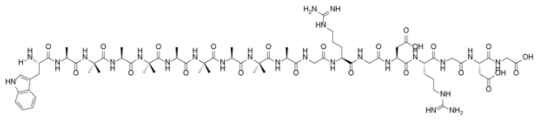

$\alpha_{4}$-(RGD)$_{2}$

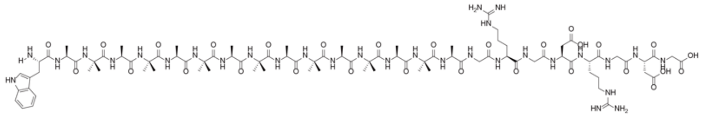

$\alpha_{7}$-(RGD)$_{2}$

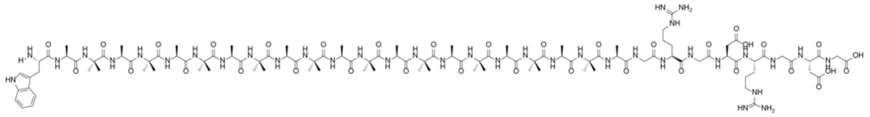

$\alpha_{10}$-(RGD)$_{2}$

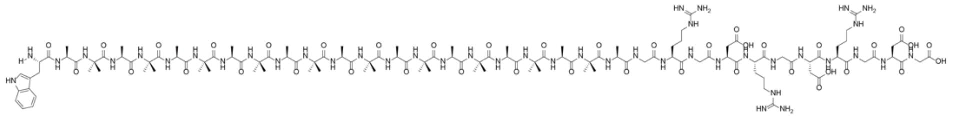

$\alpha_{10}$-(RGD)$_{3}$

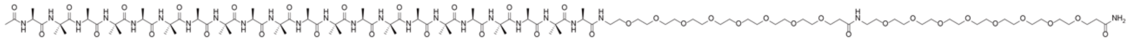

$\alpha_{10}$-PEG$_{16}$

**b**

***Linear rod-coil building blocks***

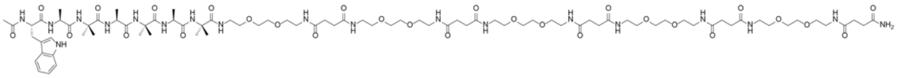

L-$\alpha_{3}$-PEG$_{10}$

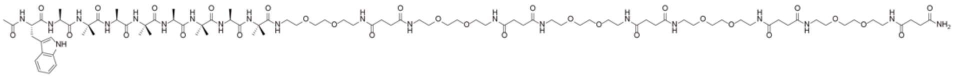

L-$\alpha_{4}$-PEG$_{10}$

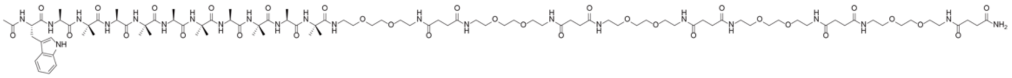

L-$\alpha_{5}$-PEG$_{10}$

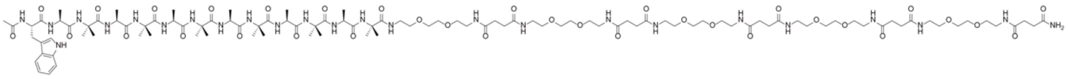

L-$\alpha_{6}$-PEG$_{10}$

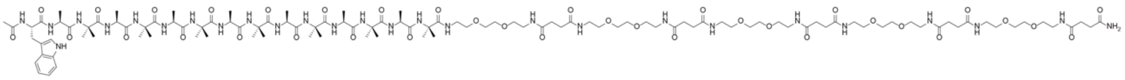

L-$\alpha_{7}$-PEG$_{10}$

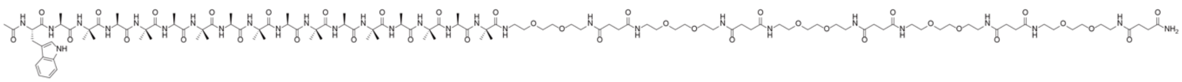

L-$\alpha_{8}$-PEG$_{10}$

**c**

***Cyclic rod-coil building blocks***

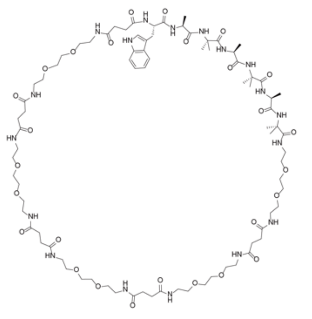

C-$\alpha_{3}$-PEG$_{10}$

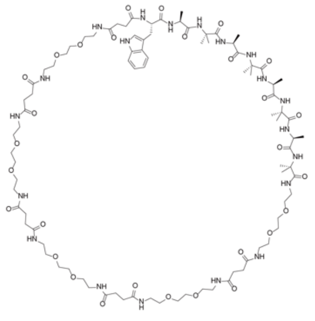

C-$\alpha_{4}$-PEG$_{10}$

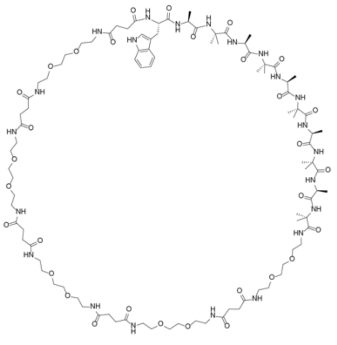

C-$\alpha_{5}$-PEG$_{10}$

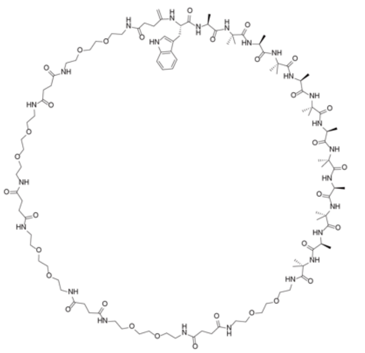

C-$\alpha_{6}$-PEG$_{10}$

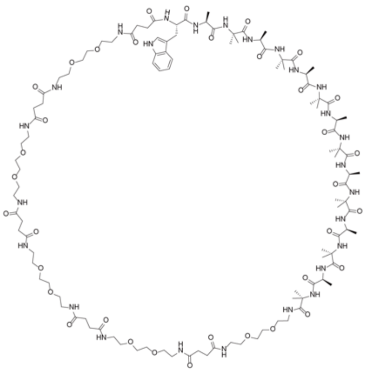

C-$\alpha_{7}$-PEG$_{10}$

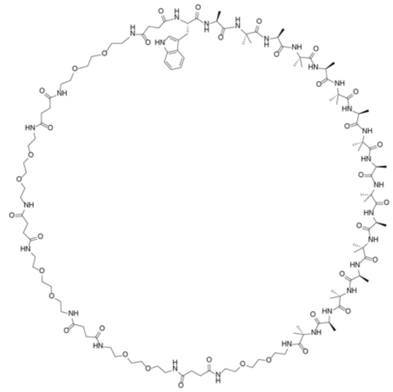

C-$\alpha_{8}$-PEG$_{10}$

**d**

***Coil-rod-coil monomers***

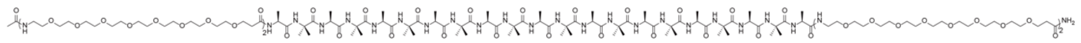

PEG$_{16}$-$\alpha_{10}$-PEG$_{16}$

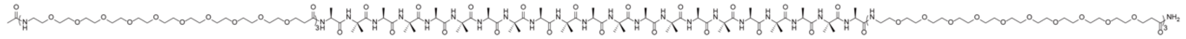

PEG$_{30}$-$\alpha_{10}$-PEG$_{30}$

**e**

***Building blocks for DNA packaging***

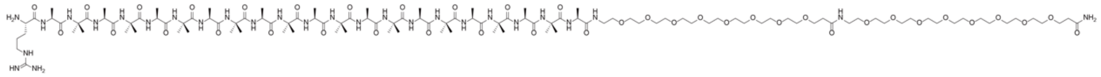

R$_{1}$-$\alpha_{10}$-PEG$_{16}$

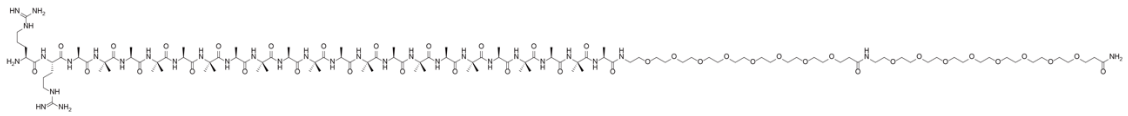

R$_{2}$-$\alpha_{10}$-PEG$_{16}$

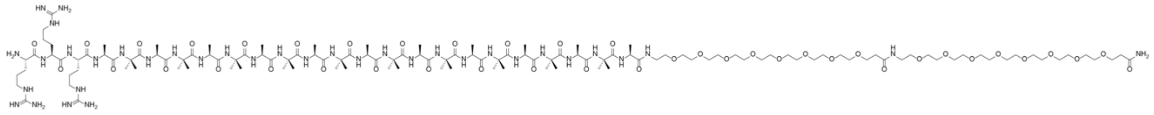

R$_{3}$-$\alpha_{10}$-PEG$_{16}$

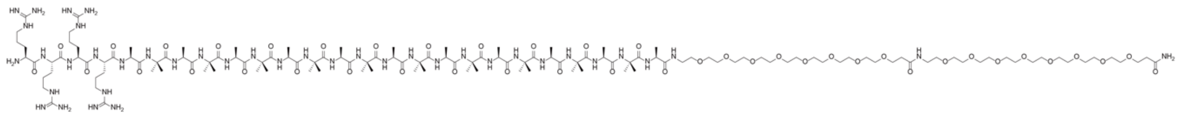

R$_{4}$-$\alpha_{10}$-PEG$_{16}$

**Supplementary Figure 1. a**–**e**, Chemical structures of the peptide-based rod-coils and coil-rod-coils used in this study.

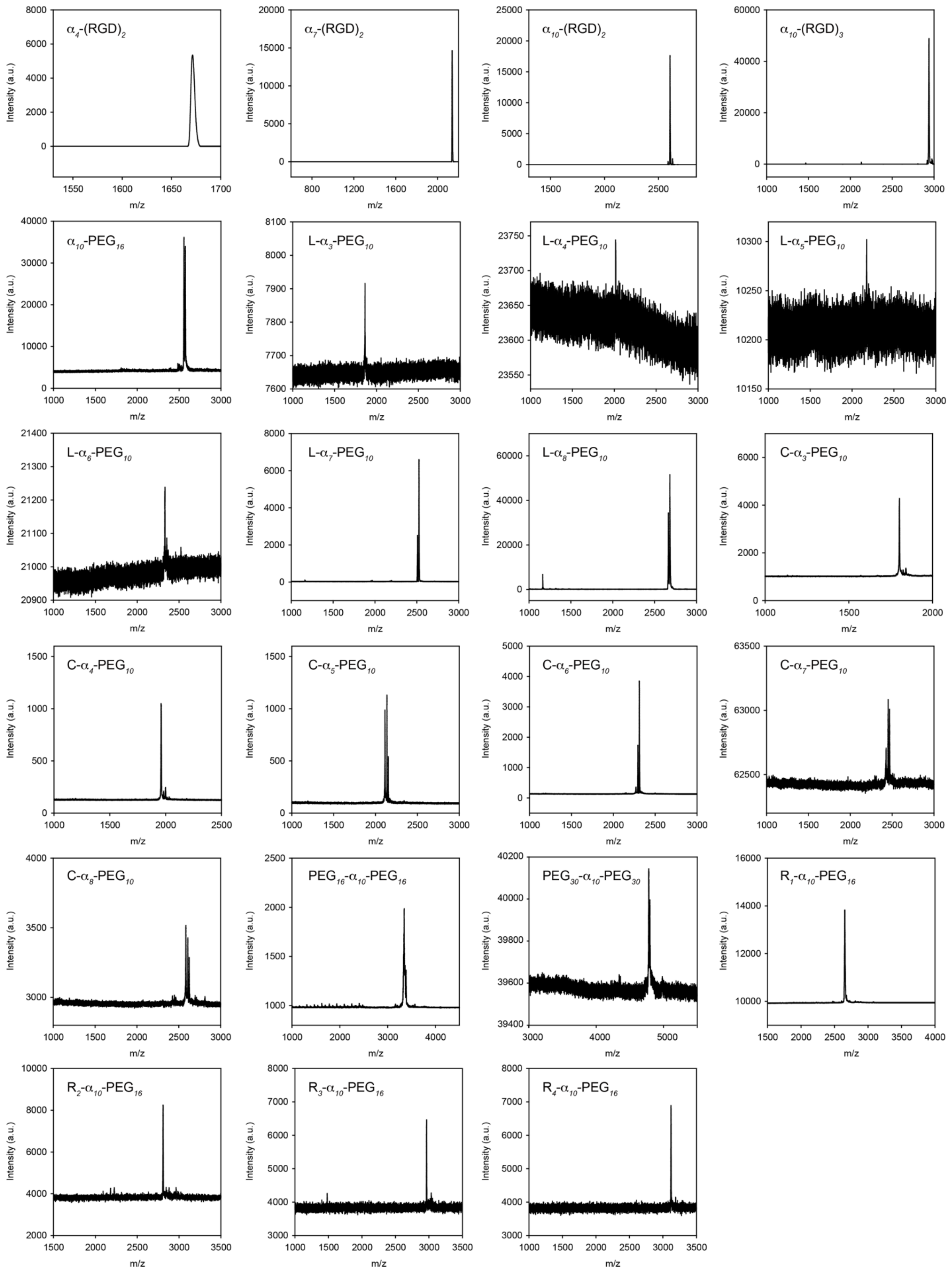


**Supplementary Figure 2.** MALDI-TOF MS spectra of the purified peptides.

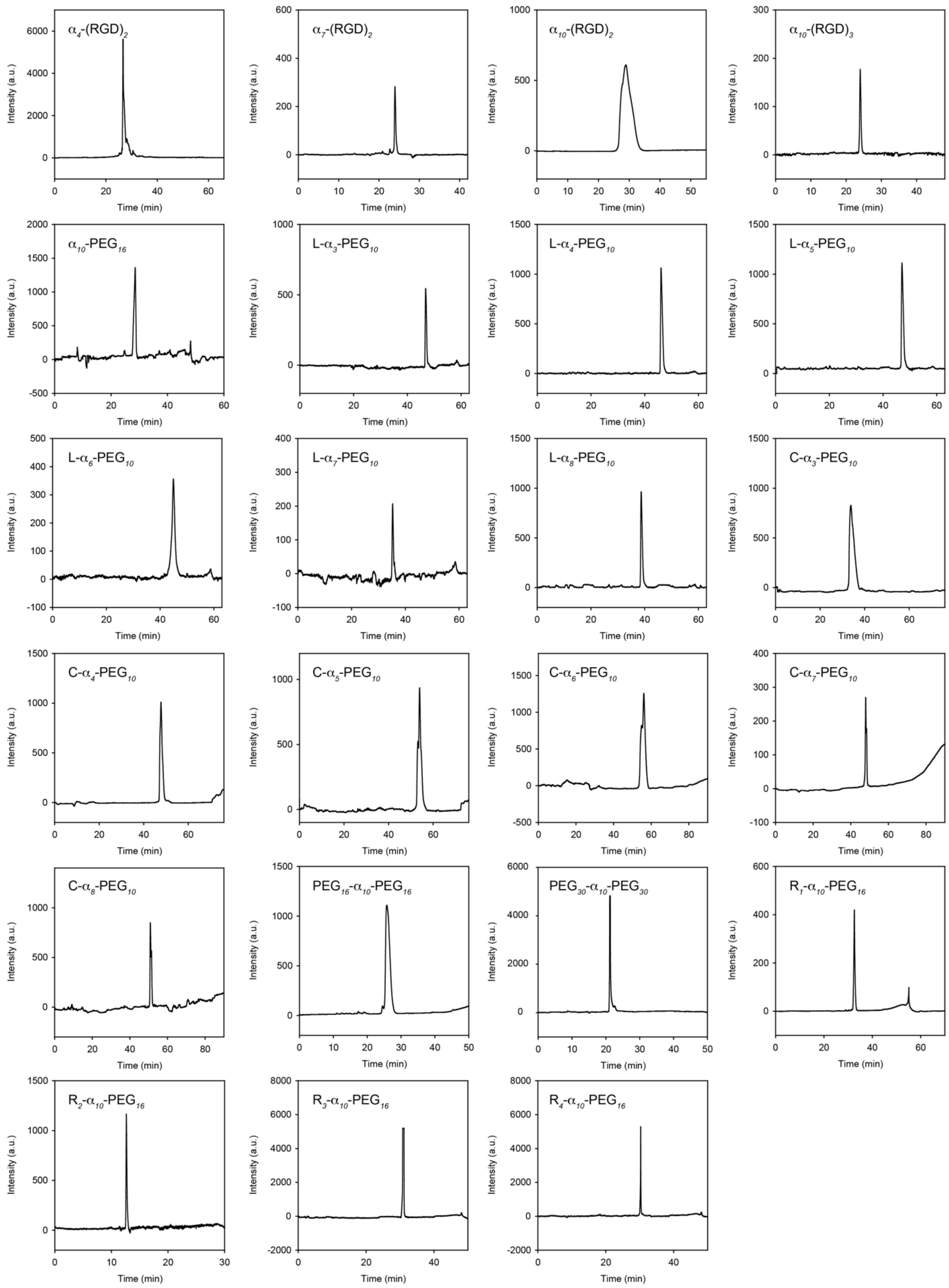


**Supplementary Figure 3.** HPLC chromatograms for the purified peptides.

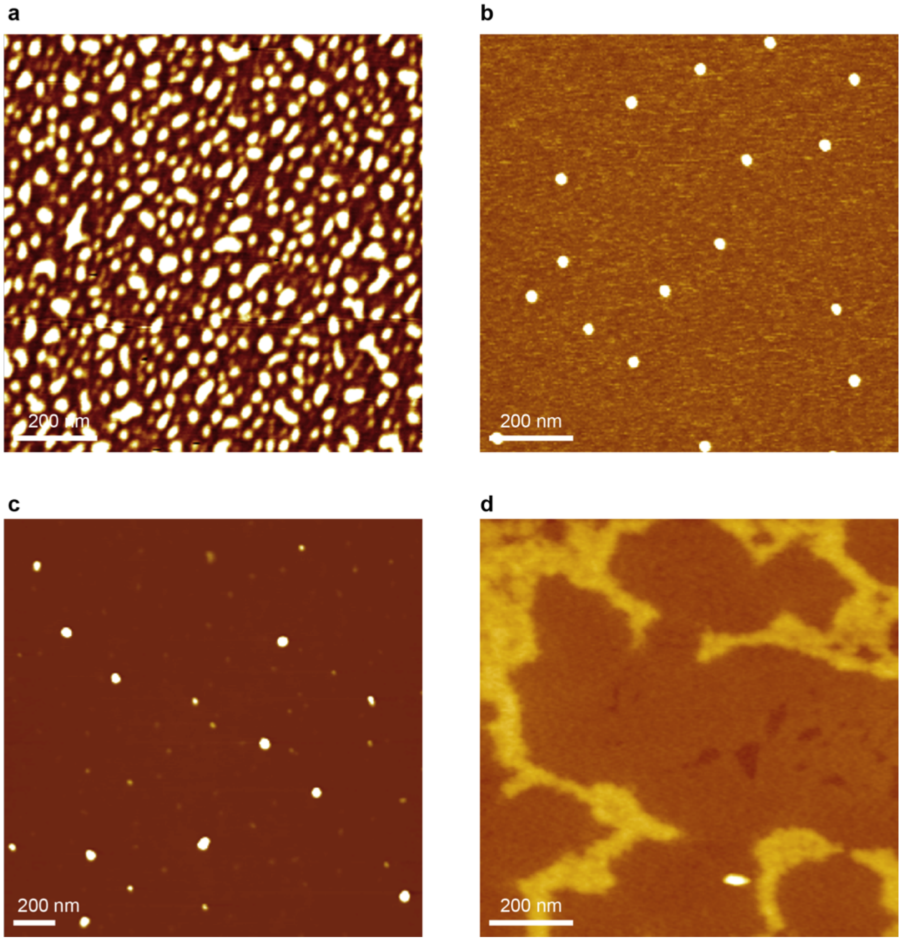


**Supplementary Figure 4.** Self-assembly behaviours of rod-coils and a coil-rod-coil. AFM images. **a,** $\alpha_{4}$-(RGD)$_{2}$. **b,** $\alpha_{7}$-(RGD)$_{2}$. **c,** $\alpha_{10}$-(RGD)$_{2}$. **d,** PEG$_{16}$-$\alpha_{10}$-PEG$_{16}$.

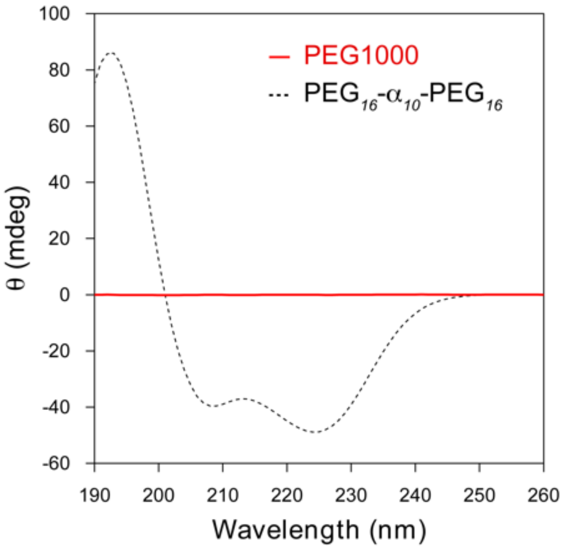


**Supplementary Figure 5.** Comparison of CD spectra between PEG1000 and PEG$_{16}$-α$_{10}$-PEG$_{16}$ at the identical experimental condition. (50 μM in water).

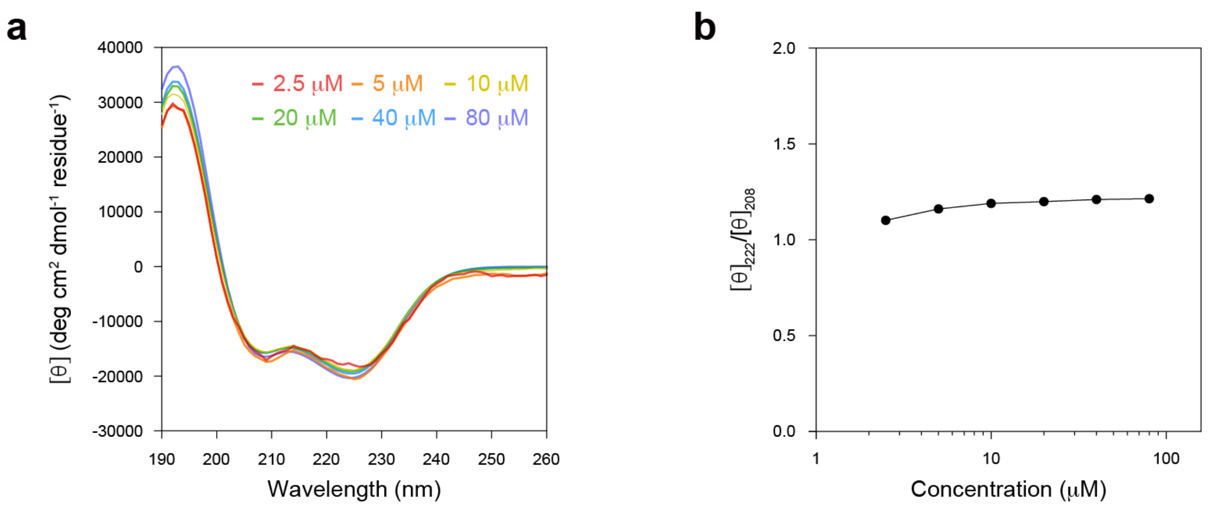


**Supplementary Figure 6.** CD data of PEG*30*-α*10*-PEG*30*. **a,** CD spectra obtained at various concentrations. **b,** Concentration independence of the helicity.

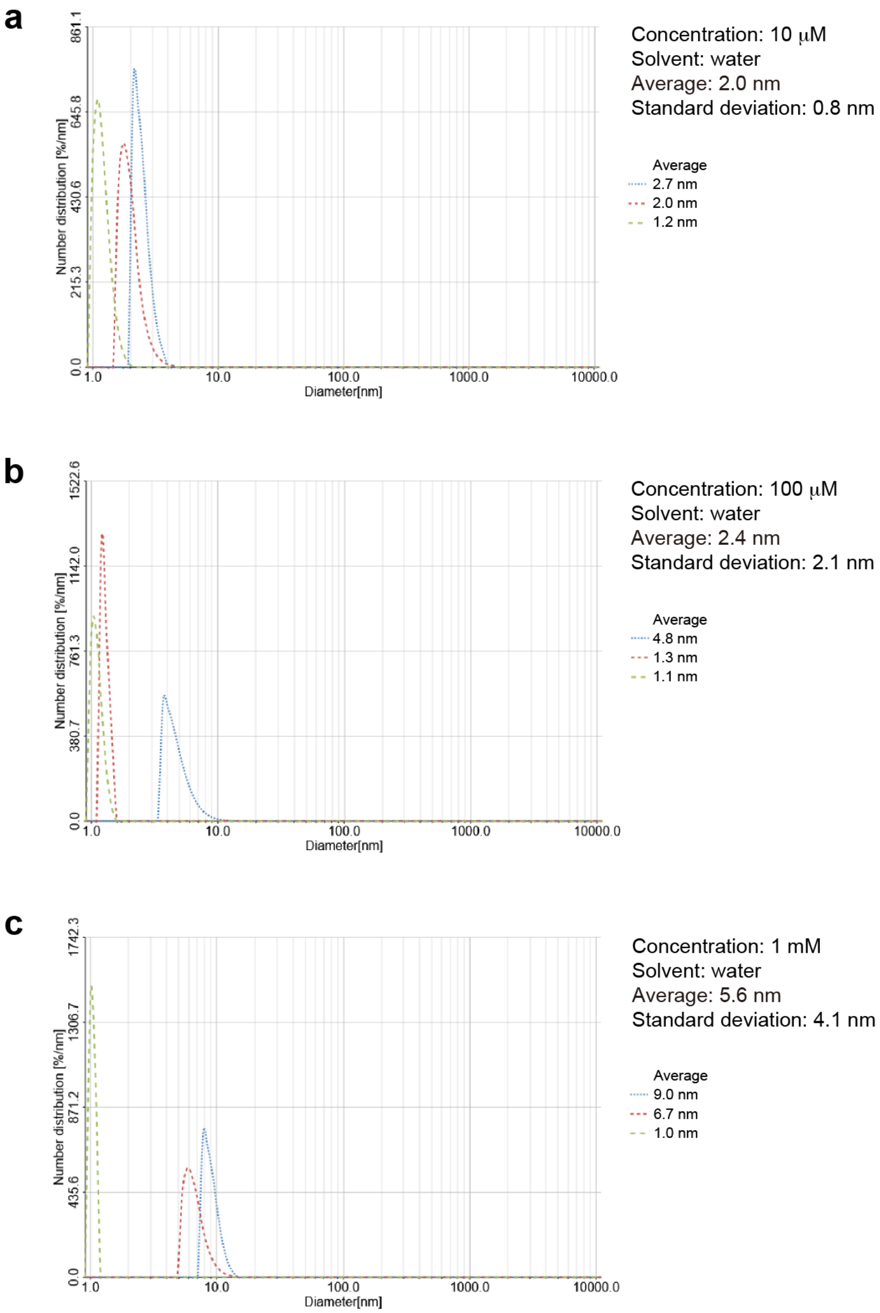


**Supplementary Figure 7.** DLS data of $PEG_{30}$-$\alpha_{10}$-$PEG_{30}$ in aqueous solution **a,** 10 μM. **b,** 100 μM. **c,** 1 mM.

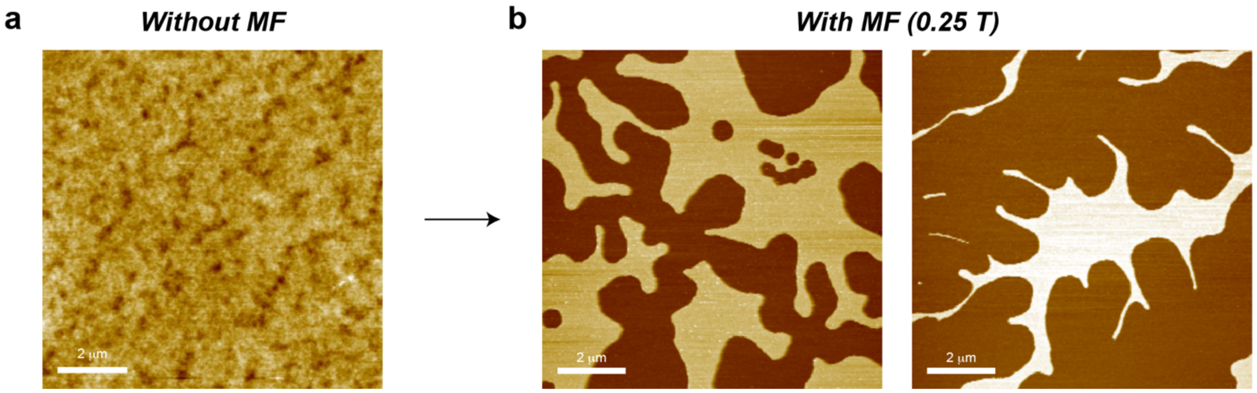


**Supplementary Figure 8.** AFM images of PEG$_{30}$-α$_{10}$-PEG$_{30}$ at 1 mM with or without magnetic field. **a,** The sample dried on mica without MF. **b,** The sample dried on mica in presence of static MF. Although discrete molecules assemblies were not formed after the MF exposure to the monomeric building block molecule, i.e., PEG$_{30}$-α$_{10}$-PEG$_{30}$, it is likely that the molecules are affected by MF and move in certain directions on the surface of mica.

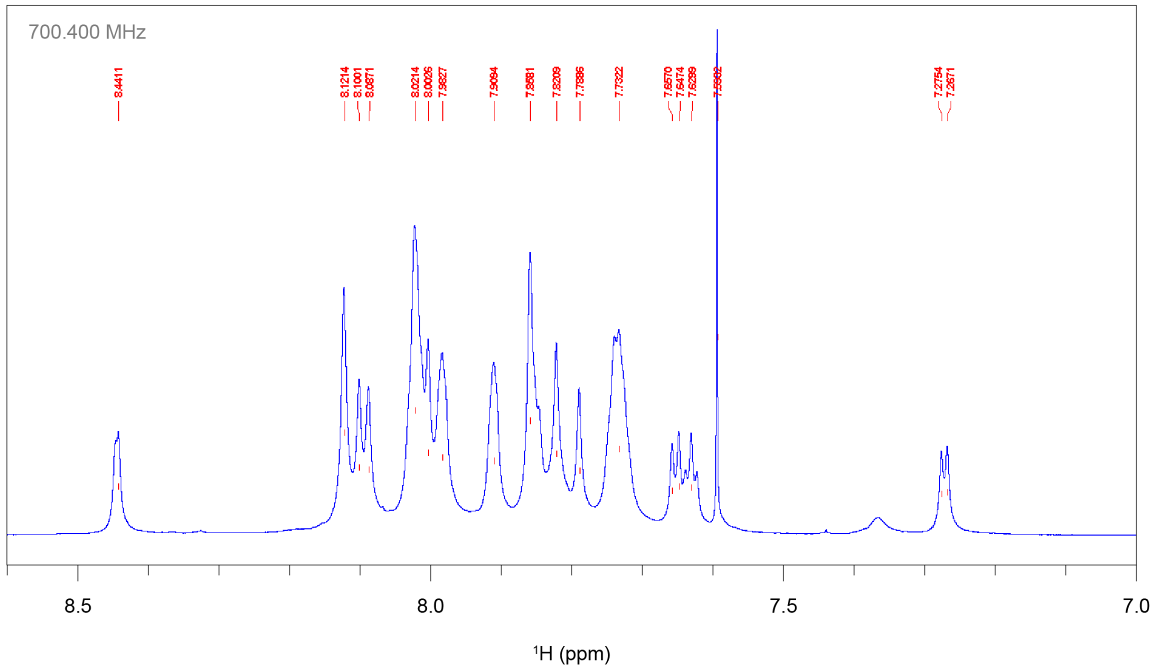


**Supplementary Figure 9.** $^1$H NMR spectrum of PEG$_{30}$-α$_{10}$-PEG$_{30}$ at 700.400 MHz (16.45 T).

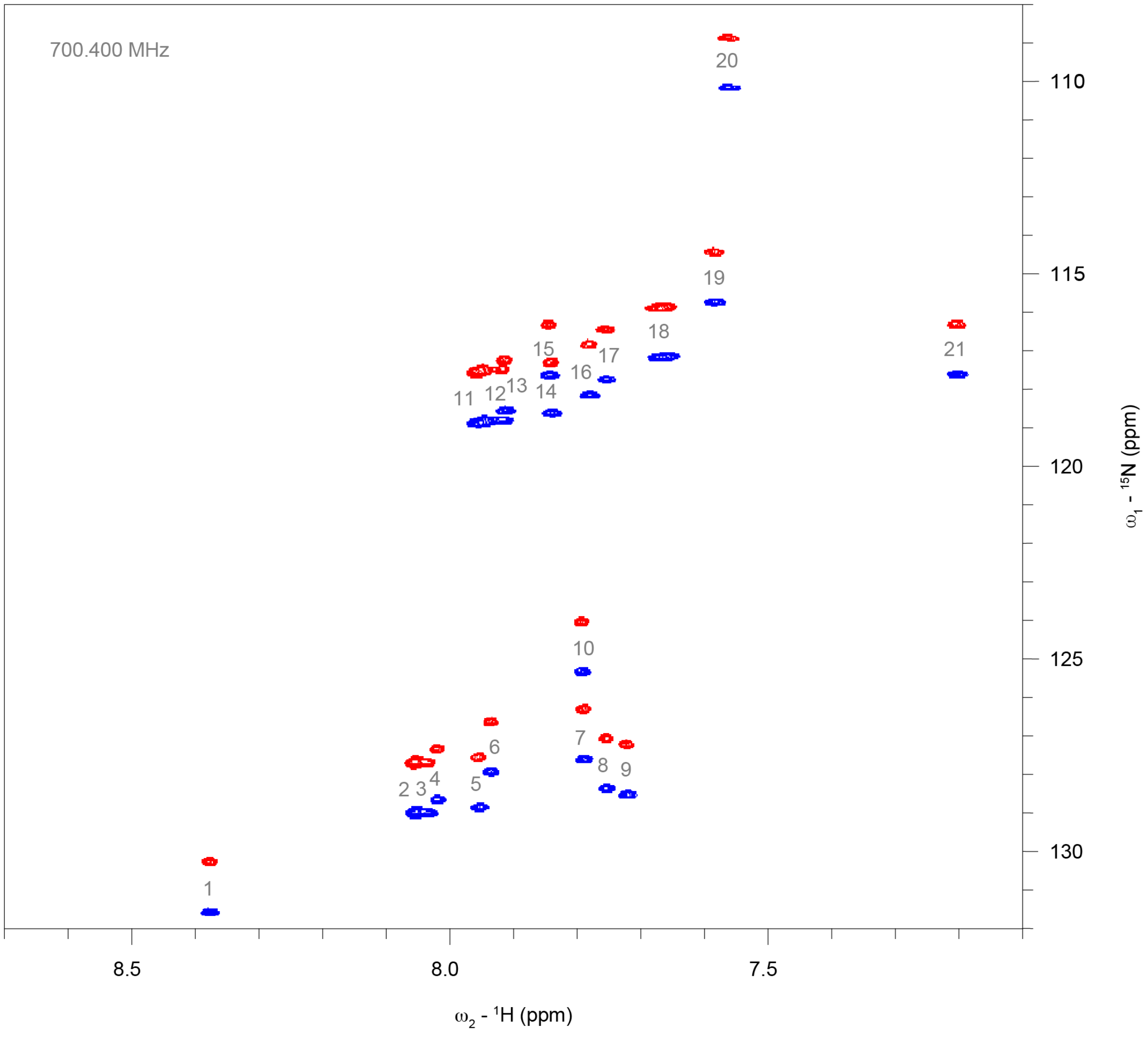


**Supplementary Figure 10.** $^{1}$H–$^{15}$N IPAP-HSQC spectrum of PEG*30*-α*10*-PEG*30* at 700.400 MHz (16.45 T).

**Supplementary table 1.** $^{1}J_{NH} + {}^{1}D_{NH}$ splittings of PEG$_{30}$-α$_{10}$-PEG$_{30}$ measured at 700.400 MHz (16.45 T).

| 700.400 MHz | $^{1}$H (ppm) | $^{1}$H (Hz) | $^{15}$N (ppm) | $^{15}$N (Hz) | $^{1}J_{NH} + {}^{1}D_{NH}$ (Hz) |
|---|---|---|---|---|---|
| 1 | 8.379 | 594.892 | 130.234 | 9246.354 | 93.149 |
| | 8.378 | 594.821 | 131.546 | 9339.503 | |
| 2 | 8.057 | 572.031 | 127.645 | 9062.540 | 93.078 |
| | 8.056 | 571.960 | 128.956 | 9155.618 | |
| 3 | 8.035 | 570.469 | 127.661 | 9063.676 | 93.007 |
| | 8.035 | 570.469 | 128.971 | 9156.683 | |
| 4 | 8.022 | 569.546 | 127.309 | 9038.684 | 92.794 |
| | 8.021 | 569.475 | 128.616 | 9131.479 | |
| 5 | 7.957 | 564.931 | 127.514 | 9053.239 | 92.794 |
| | 7.955 | 564.789 | 128.821 | 9146.033 | |
| 6 | 7.938 | 563.582 | 126.592 | 8987.779 | 92.510 |
| | 7.937 | 563.511 | 127.895 | 9080.289 | |
| 7 | 7.793 | 553.287 | 126.271 | 8964.988 | 92.581 |
| | 7.791 | 553.145 | 127.575 | 9057.570 | |
| 8 | 7.757 | 550.731 | 127.027 | 9018.663 | 92.084 |
| | 7.756 | 550.660 | 128.324 | 9110.747 | |
| 9 | 7.724 | 548.389 | 127.182 | 9029.668 | 92.581 |
| | 7.723 | 548.318 | 128.486 | 9122.249 | |
| 10 | 7.796 | 553.500 | 123.999 | 8803.681 | 92.084 |
| | 7.794 | 553.358 | 125.296 | 8895.765 | |
| 11 | 7.958 | 565.002 | 117.523 | 8343.898 | 93.717 |
| | 7.957 | 564.931 | 118.843 | 8437.615 | |
| 12 | 7.920 | 562.304 | 117.445 | 8338.360 | 94.001 |
| | 7.919 | 562.233 | 118.769 | 8432.361 | |
| 13 | 7.916 | 562.020 | 117.202 | 8321.108 | 93.717 |
| | 7.914 | 561.878 | 118.522 | 8414.825 | |
| 14 | 7.843 | 556.837 | 117.266 | 8325.651 | 93.291 |
| | 7.842 | 556.766 | 118.580 | 8418.943 | |
| 15 | 7.847 | 557.121 | 116.276 | 8255.363 | 93.646 |
| | 7.845 | 556.979 | 117.595 | 8349.010 | |
| 16 | 7.784 | 552.648 | 116.804 | 8292.850 | 93.149 |
| | 7.782 | 552.506 | 118.116 | 8386.000 | |
| 17 | 7.757 | 550.731 | 116.408 | 8264.735 | 92.510 |
| | 7.756 | 550.660 | 117.711 | 8357.246 | |
| 18 | 7.671 | 544.626 | 115.840 | 8224.408 | 92.439 |
| | 7.669 | 544.484 | 117.142 | 8316.848 | |
| 19 | 7.588 | 538.733 | 114.392 | 8121.603 | 92.723 |
| | 7.586 | 538.591 | 115.698 | 8214.327 | |
| 20 | 7.565 | 537.100 | 108.835 | 7727.067 | 92.652 |
| | 7.564 | 537.029 | 110.140 | 7819.720 | |
| 21 | 7.207 | 511.683 | 116.274 | 8255.221 | 92.226 |
| | 7.206 | 511.612 | 117.573 | 8347.448 | |
| Average | | | | | 92.892 |

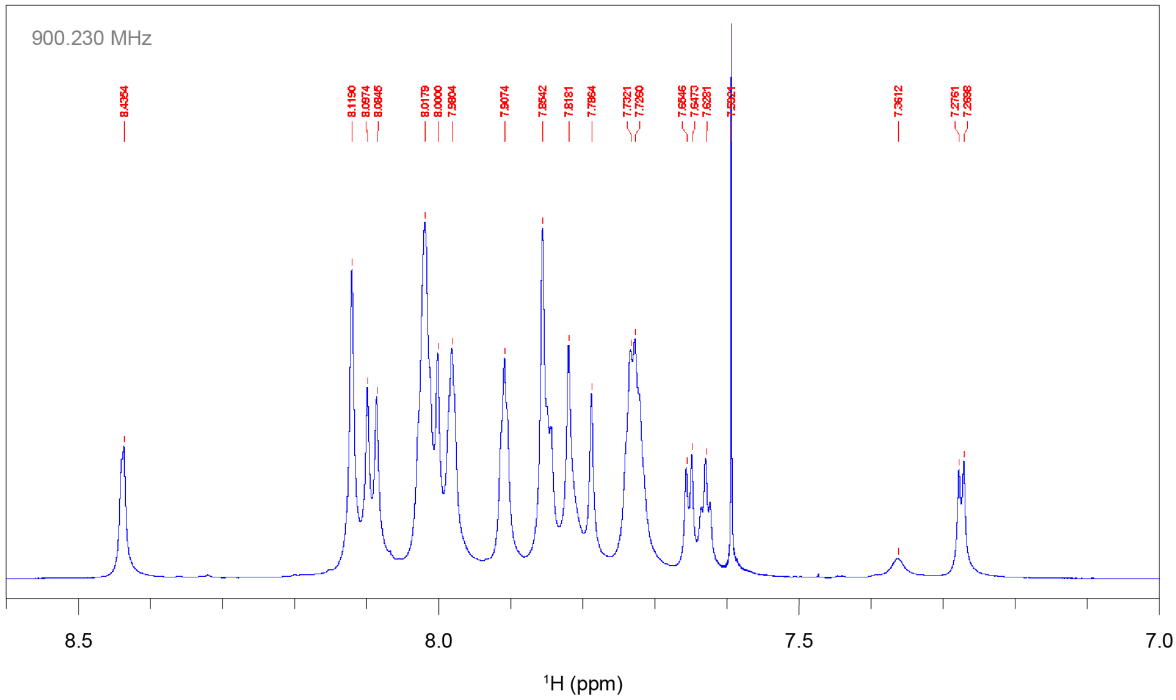


**Supplementary Figure 11.** $^1$H NMR spectrum of PEG$_{30}$-α$_{10}$-PEG$_{30}$ at 900.230 MHz (21.14 T).

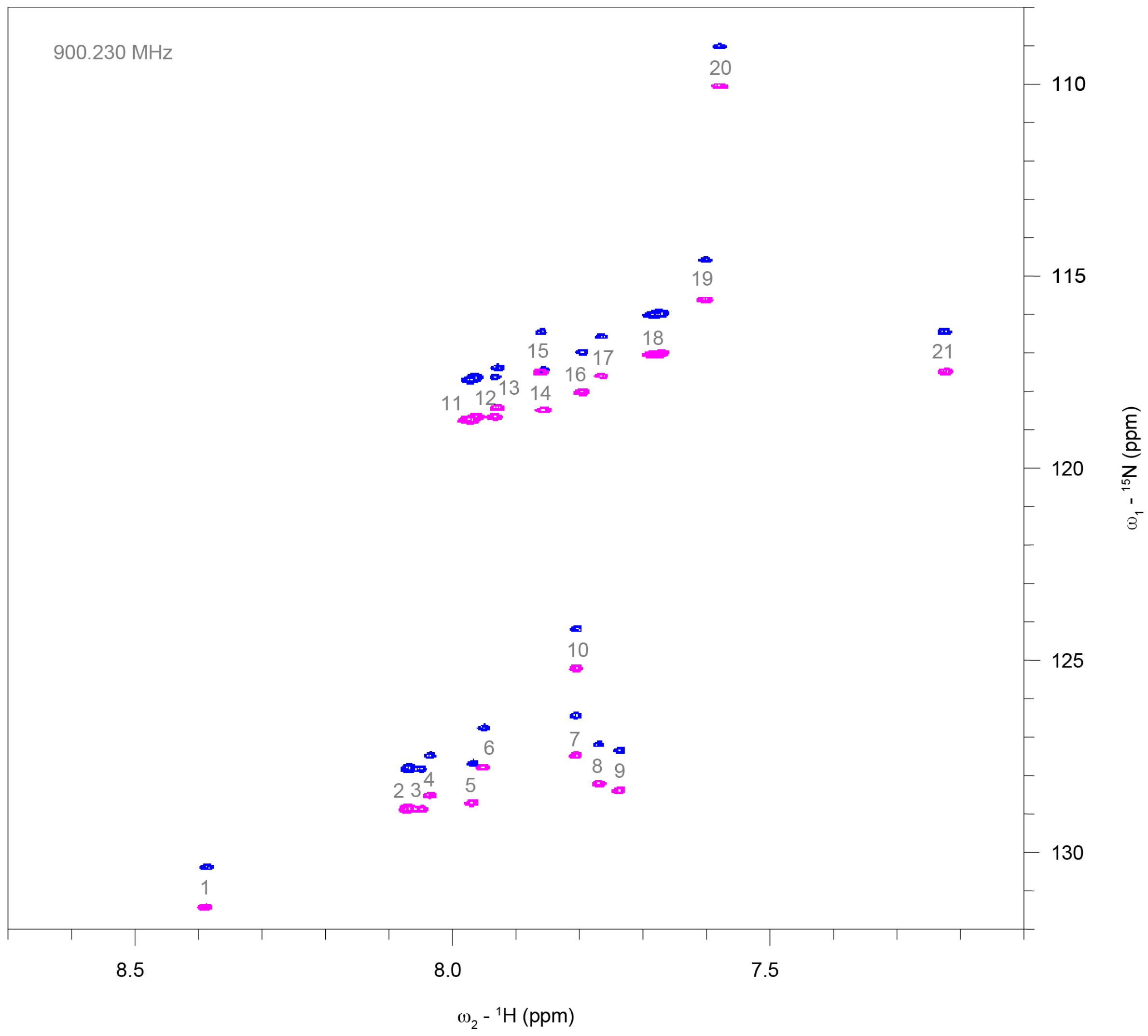


**Supplementary Figure 12.** $^{1}H$–$^{15}N$ IPAP-HSQC spectrum of PEG*30*-α*10*-PEG*30* at 900.230 MHz (21.14 T).

**Supplementary table 2.** $^{1}J_{NH} + {}^{1}D_{NH}$ splittings of PEG$_{30}$-α$_{10}$-PEG$_{30}$ measured at 900.230 MHz (21.14 T).

| 900.230 MHz | $^{1}$H (ppm) | $^{1}$H (Hz) | $^{15}$N (ppm) | $^{15}$N (Hz) | $^{1}J_{NH} + {}^{1}D_{NH}$ (Hz) |
|---|---|---|---|---|---|
| 1 | 8.386 | 765.256 | 130.351 | 11895.050 | 95.087 |
| | 8.388 | 765.439 | 131.393 | 11990.137 | |
| 2 | 8.068 | 736.237 | 127.769 | 11659.432 | 96.364 |
| | 8.069 | 736.329 | 128.825 | 11755.797 | |
| 3 | 8.046 | 734.230 | 127.798 | 11662.079 | 94.813 |
| | 8.047 | 734.321 | 128.837 | 11756.892 | |
| 4 | 8.033 | 733.043 | 127.439 | 11629.319 | 94.813 |
| | 8.034 | 733.135 | 128.478 | 11724.131 | |
| 5 | 7.967 | 727.021 | 127.641 | 11647.752 | 94.995 |
| | 7.968 | 727.112 | 128.682 | 11742.747 | |
| 6 | 7.949 | 725.378 | 126.715 | 11563.251 | 95.087 |
| | 7.950 | 725.469 | 127.757 | 11658.337 | |
| 7 | 7.804 | 712.146 | 126.400 | 11534.506 | 94.904 |
| | 7.805 | 712.237 | 127.440 | 11629.410 | |
| 8 | 7.768 | 708.861 | 127.150 | 11602.946 | 94.083 |
| | 7.768 | 708.861 | 128.181 | 11697.029 | |
| 9 | 7.736 | 705.941 | 127.311 | 11617.638 | 94.813 |
| | 7.736 | 705.941 | 128.350 | 11712.451 | |
| 10 | 7.803 | 712.055 | 124.138 | 11328.089 | 94.083 |
| | 7.804 | 712.146 | 125.169 | 11422.172 | |
| 11 | 7.972 | 727.477 | 117.658 | 10736.763 | 95.087 |
| | 7.972 | 727.477 | 118.700 | 10831.850 | |
| 12 | 7.932 | 723.827 | 117.578 | 10729.463 | 95.360 |
| | 7.932 | 723.827 | 118.623 | 10824.823 | |
| 13 | 7.928 | 723.462 | 117.335 | 10707.288 | 95.360 |
| | 7.928 | 723.462 | 118.380 | 10802.649 | |
| 14 | 7.854 | 716.709 | 117.394 | 10712.672 | 95.452 |
| | 7.855 | 716.800 | 118.440 | 10808.124 | |
| 15 | 7.858 | 717.074 | 116.406 | 10622.513 | 95.634 |
| | 7.859 | 717.165 | 117.454 | 10718.147 | |
| 16 | 7.794 | 711.234 | 116.933 | 10670.604 | 95.178 |
| | 7.795 | 711.325 | 117.976 | 10765.782 | |
| 17 | 7.763 | 708.405 | 116.525 | 10633.372 | 93.992 |
| | 7.764 | 708.496 | 117.555 | 10727.364 | |
| 18 | 7.679 | 700.739 | 115.952 | 10581.084 | 94.630 |
| | 7.680 | 700.831 | 116.989 | 10675.714 | |
| 19 | 7.600 | 693.530 | 114.526 | 10450.956 | 94.630 |
| | 7.601 | 693.622 | 115.563 | 10545.586 | |
| 20 | 7.578 | 691.523 | 108.969 | 9943.857 | 94.083 |
| | 7.578 | 691.523 | 110.000 | 10037.940 | |
| 21 | 7.224 | 659.219 | 116.401 | 10622.057 | 94.448 |
| | 7.223 | 659.128 | 117.436 | 10716.505 | |
| Average | | | | | 94.900 |

**Supplementary table 3.** Experimental residual dipolar couplings (RDCs; $^{1}D_{NH, exp}$) of PEG*30*-α*10*-PEG*30*. ($^{1}D_{NH, exp}$) = $(^{1}J_{NH} + {}^{1}D_{NH})^{(21.14\ T)} - (^{1}J_{NH} + {}^{1}D_{NH})^{(16.45\ T)}$.

| | $(^{1}J_{NH} + {}^{1}D_{NH})^{21.14\ T}$ | $(^{1}J_{NH} + {}^{1}D_{NH})^{16.45\ T}$ | $^{1}D_{NH, exp}$ |
|---|---|---|---|
| 1 | 95.087 | 93.149 | 1.937 |
| 2 | 96.364 | 93.078 | 3.286 |
| 3 | 94.813 | 93.007 | 1.806 |
| 4 | 94.813 | 92.794 | 2.019 |
| 5 | 94.995 | 92.794 | 2.201 |
| 6 | 95.087 | 92.510 | 2.576 |
| 7 | 94.904 | 92.581 | 2.323 |
| 8 | 94.083 | 92.084 | 1.998 |
| 9 | 94.813 | 92.581 | 2.232 |
| 10 | 94.083 | 92.084 | 1.998 |
| 11 | 95.087 | 93.717 | 1.369 |
| 12 | 95.360 | 94.001 | 1.359 |
| 13 | 95.360 | 93.717 | 1.643 |
| 14 | 95.452 | 93.291 | 2.160 |
| 15 | 95.634 | 93.646 | 1.988 |
| 16 | 95.178 | 93.149 | 2.029 |
| 17 | 93.992 | 92.510 | 1.481 |
| 18 | 94.630 | 92.439 | 2.191 |
| 19 | 94.630 | 92.723 | 1.907 |
| 20 | 94.083 | 92.652 | 1.430 |
| 21 | 94.448 | 92.226 | 2.221 |
| Average | 94.900 | 92.892 | 2.007 |

**Supplementary Table 4.** Conformational properties of the linear and cyclic rod-coils as determined by CD spectroscopy.

| Name | $[\theta]_{222}/[\theta]_{208}$ |
|---|---|
| L-$\alpha_3$-PEG$_{10}$ | n.d.[a] |
| L-$\alpha_4$-PEG$_{10}$ | n.d. |
| L-$\alpha_5$-PEG$_{10}$ | 0.84 |
| L-$\alpha_6$-PEG$_{10}$ | 0.88 |
| L-$\alpha_7$-PEG$_{10}$ | 1.05 |
| L-$\alpha_8$-PEG$_{10}$ | 1.10 |
| C-$\alpha_3$-PEG$_{10}$ | n.d. |
| C-$\alpha_4$-PEG$_{10}$ | n.d. |
| C-$\alpha_5$-PEG$_{10}$ | 0.93 |
| C-$\alpha_6$-PEG$_{10}$ | 0.95 |
| C-$\alpha_7$-PEG$_{10}$ | 1.01 |
| C-$\alpha_8$-PEG$_{10}$ | 1.10 |

[a] n.d = not determined

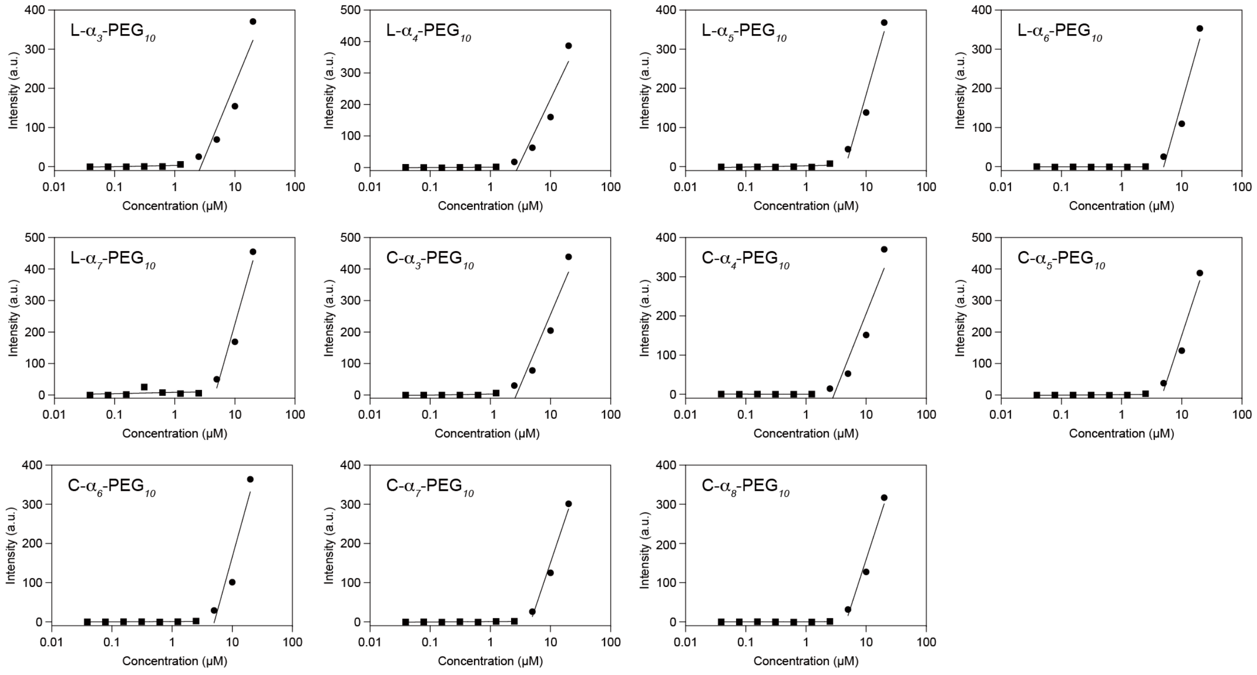


**Supplementary Figure 13.** Critical aggregation concentration (CAC) determination. CAC was calculated by plotting the changes in tryptophan fluorescence as a function of peptide concentrations.[1] The plot of tryptophan fluorescence intensity at 352 nm as a function of peptide concentrations (log scale). The intersection point of the extrapolated linear regression lines was used to calculate the CAC.

**Supplementary Table 5.** Critical aggregation concentrations (CACs).

| Peptide | CAC[a] (μM) |
|---|---|
| L-$\alpha_3$-PEG$_{10}$ | 1.56 |
| L-$\alpha_4$-PEG$_{10}$ | 1.58 |
| L-$\alpha_5$-PEG$_{10}$ | 1.95 |
| L-$\alpha_6$-PEG$_{10}$ | 2.01 |
| L-$\alpha_7$-PEG$_{10}$ | 1.98 |
| L-$\alpha_8$-PEG$_{10}$ | n.d |
| C-$\alpha_3$-PEG$_{10}$ | 1.55 |
| C-$\alpha_4$-PEG$_{10}$ | 1.60 |
| C-$\alpha_5$-PEG$_{10}$ | 1.97 |
| C-$\alpha_6$-PEG$_{10}$ | 2.03 |
| C-$\alpha_7$-PEG$_{10}$ | 1.96 |
| C-$\alpha_8$-PEG$_{10}$ | 1.95 |

[a] CACs were determined by the concentration dependent changes in tryptophan fluorescence emission.[1] [b] n.d = not determined due to the limited solubility in aqueous solution.

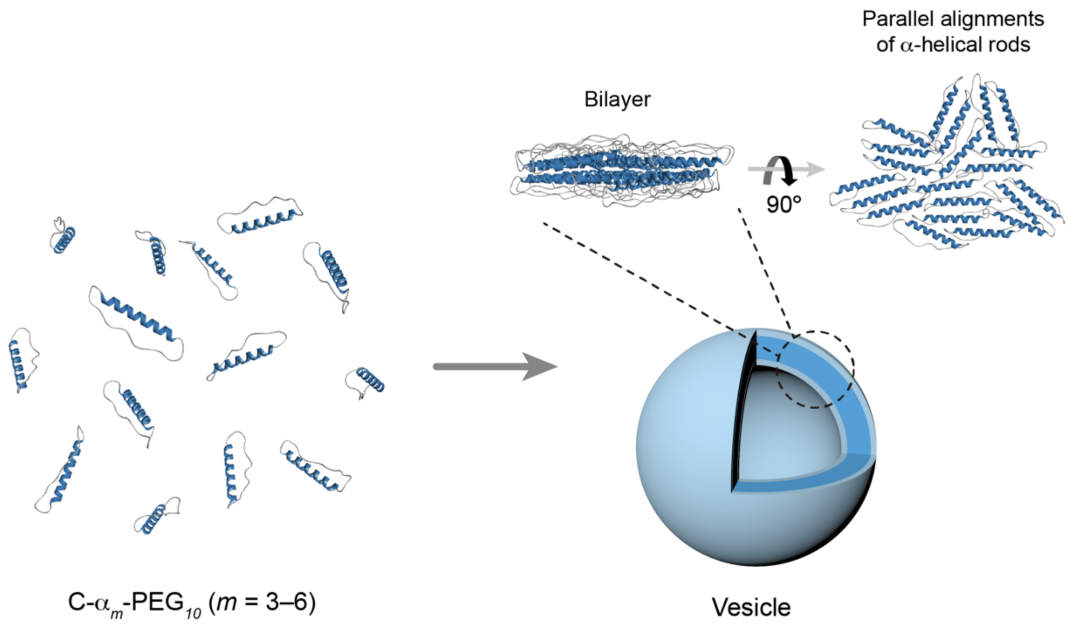


**Supplementary Figure 14.** Schematic model of vesicles formed by the self-assembly of C-α$_m$-PEG$_{10}$ (m = 3–6).

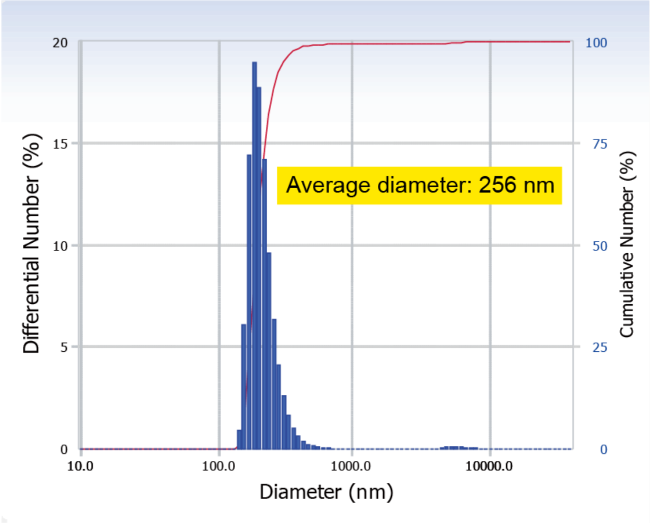


**Supplementary Figure 15.** DLS data of C-$\alpha_{7}$-PEG$_{10}$.

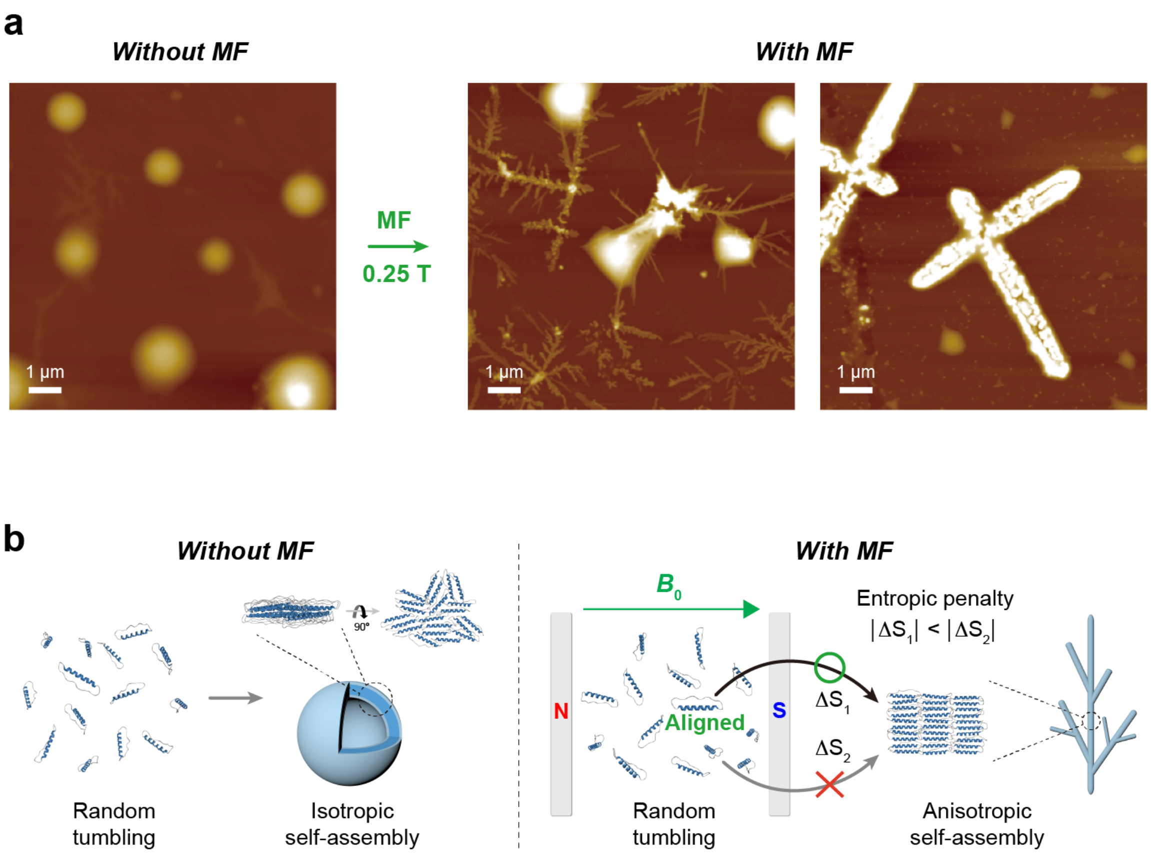


**Supplementary Figure 16.** Effect of magnetic field (MF) on the self-assembly of C-$\alpha_7$-PEG$_{10}$. **a,** AFM images before and after the MF treatment. **b,** Model of self-assembly processes in the absence or presence of the magnetic field.

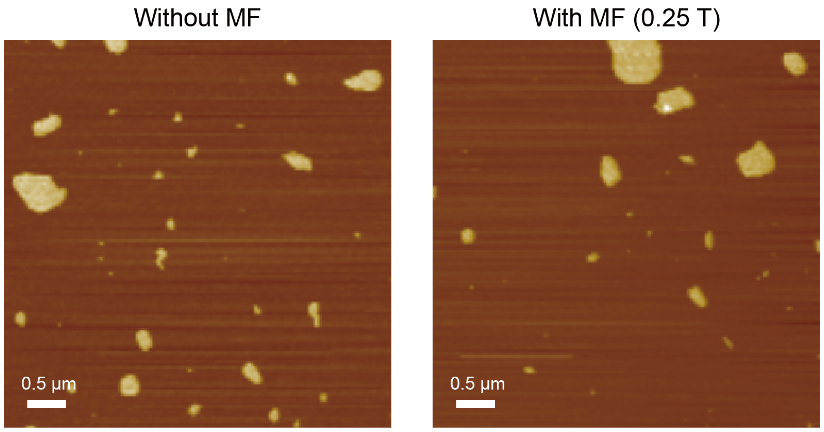


**Supplementary Figure 17.** Magnetic effect on the self-assembly of C-$\alpha_7$-PEG$_{10}$ at a concentration below CAC. AFM images. [C-$\alpha_7$-PEG$_{10}$] = 156 nM.

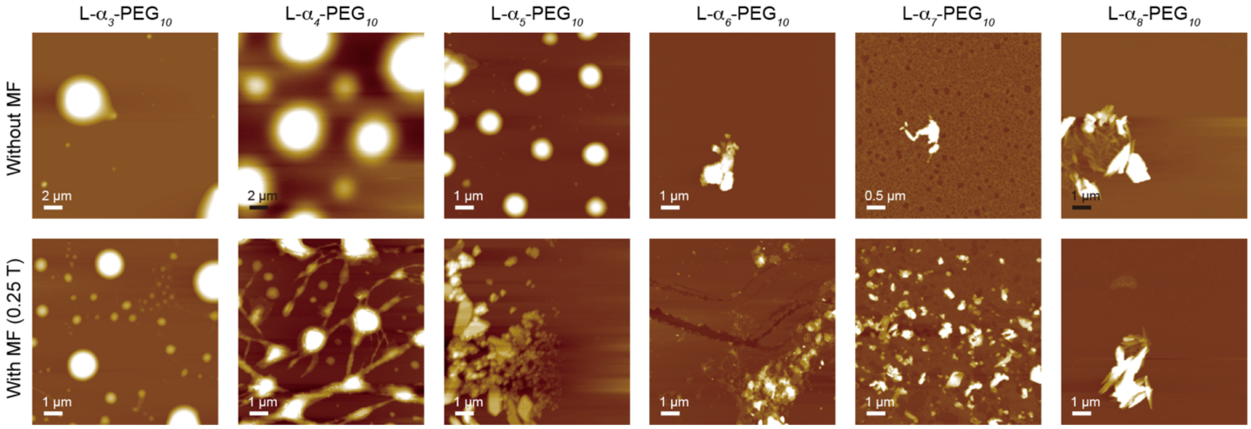


**Supplementary Figure 18.** Effect of MF (0.25 T) on the self-assembly of L-$\alpha_m$-PEG$_{10}$ ($m$ = 3–8).

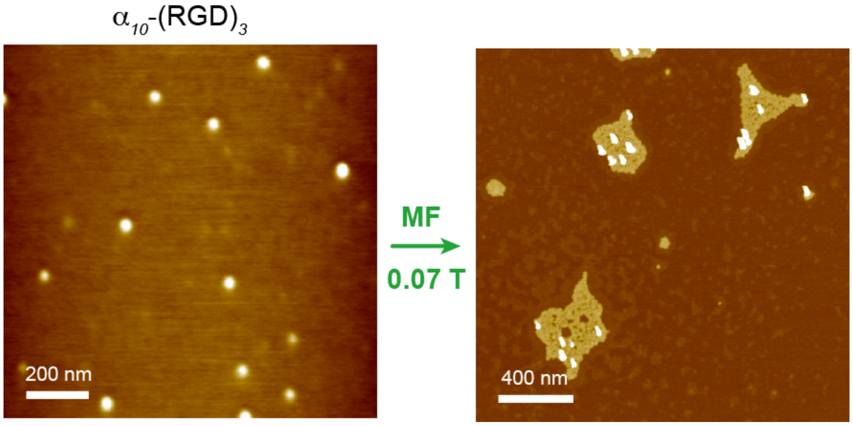


**Supplementary Figure 19.** Self-assembly of $\alpha_{10}$-(RGD)$_3$ in the absence (left) or presence (right) of the magnetic field.

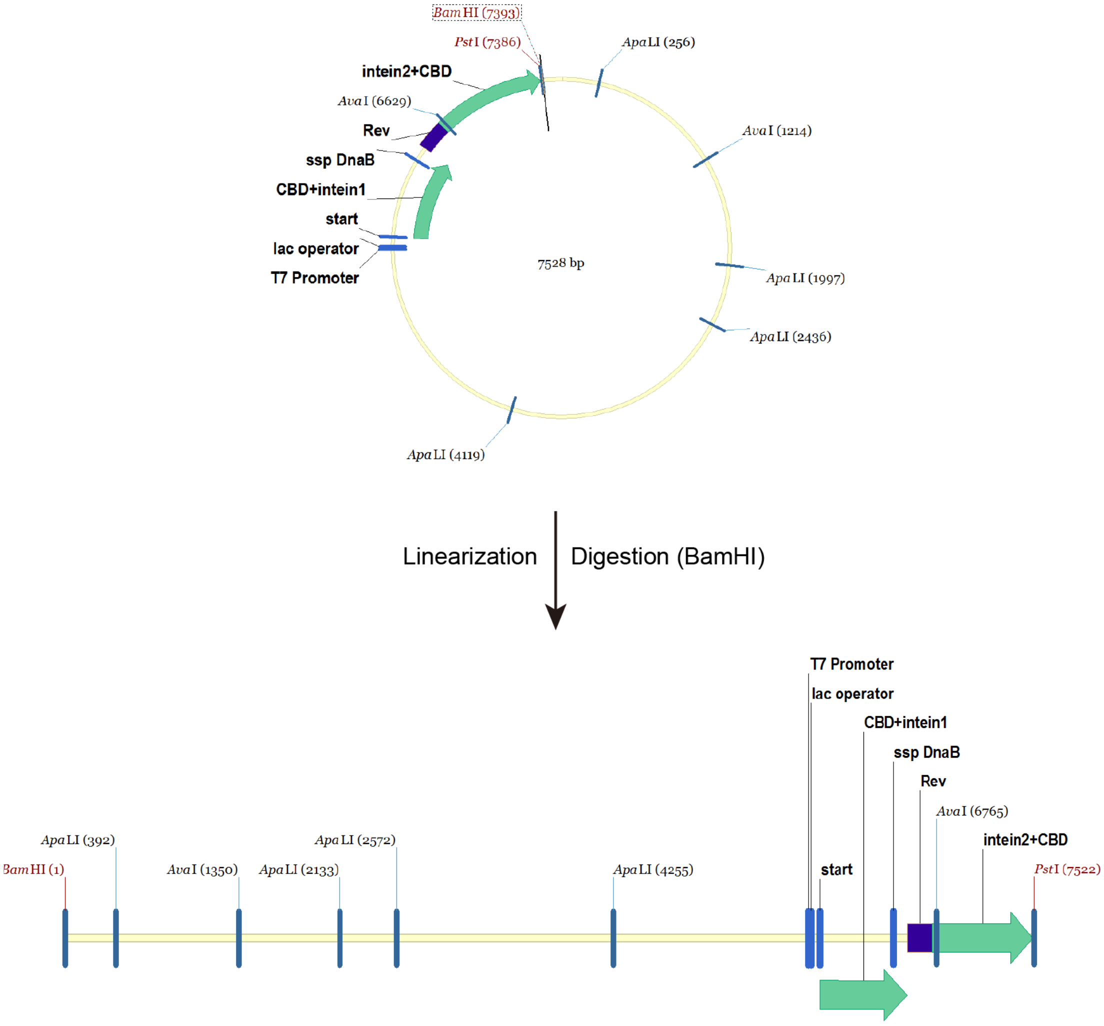


**Supplementary Figure 20.** Linearization of a plasmid DNA used in this study. The plasmid DNA was digested at the Bam HI site.

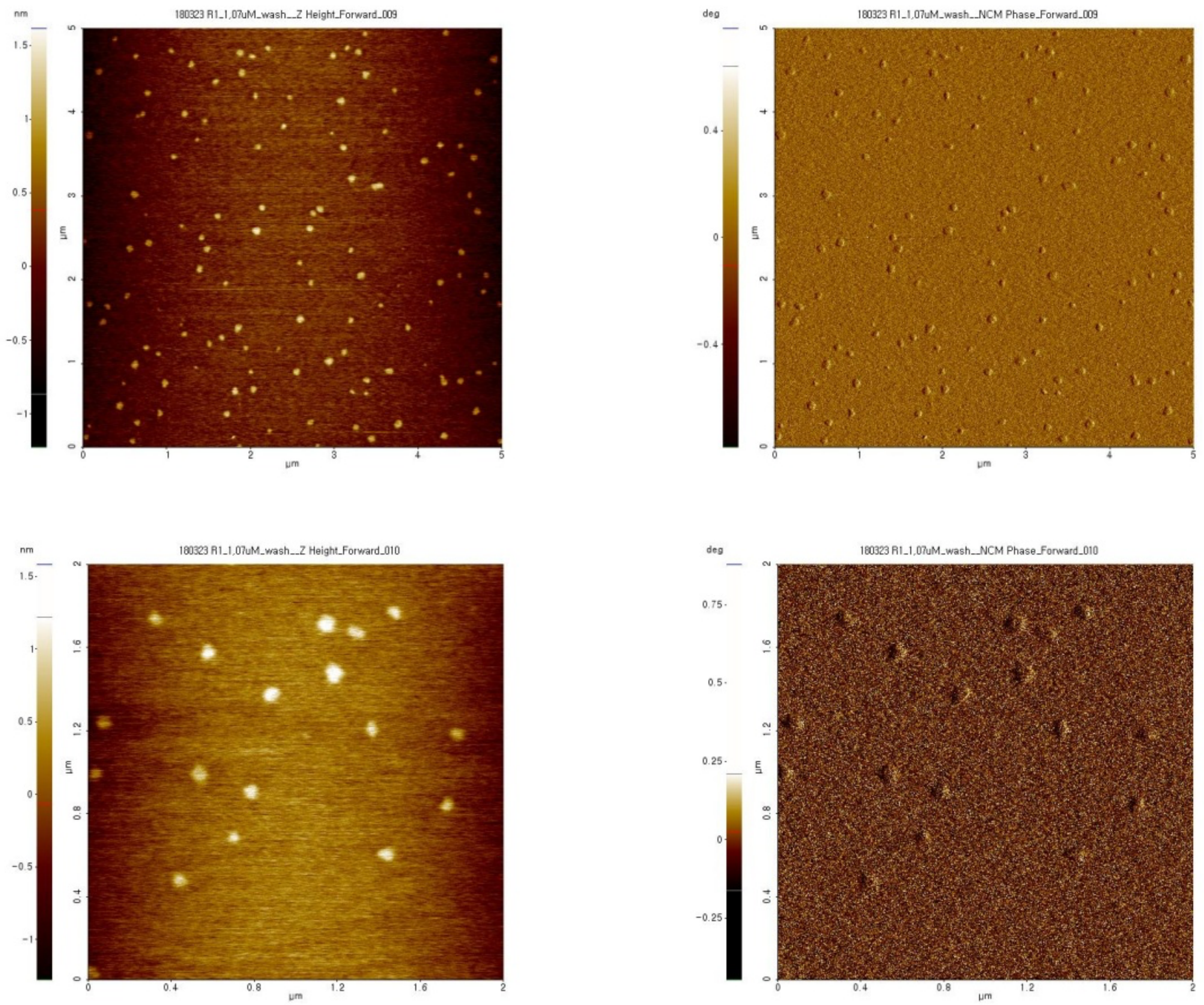


**Supplementary Figure 21.** Spherical nanostructures formed by the self-assembly of R$_{1}$-α$_{10}$-PEG$_{16}$.

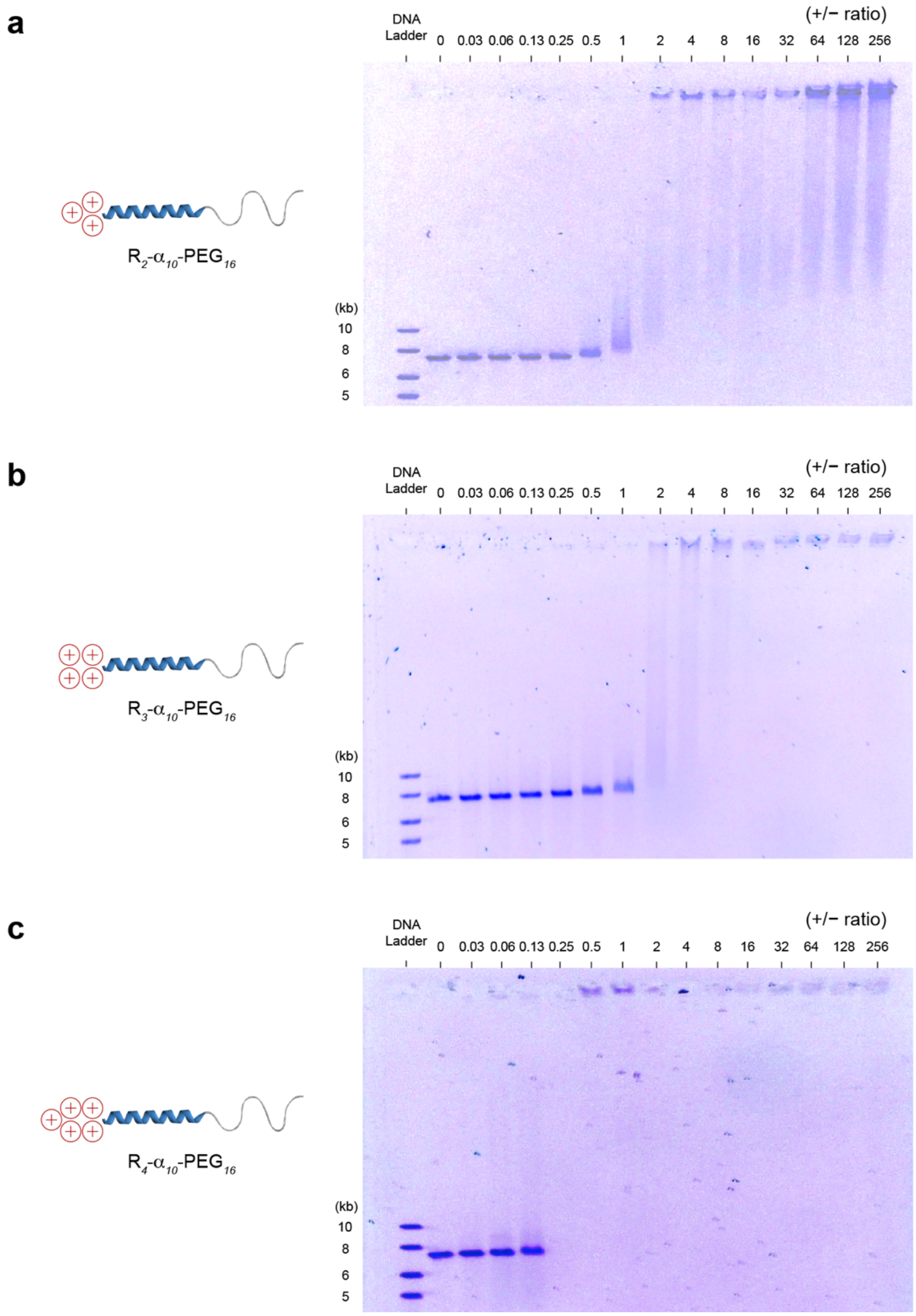


**Supplementary Figure 22.** EMSA of the linear plasmid DNA with the building blocks having 3–5 positive charges. **a,** $R_2$-$\alpha_{10}$-PEG$_{16}$, **b,** $R_3$-$\alpha_{10}$-PEG$_{16}$, **c,** $R_4$-$\alpha_{10}$-PEG$_{16}$.

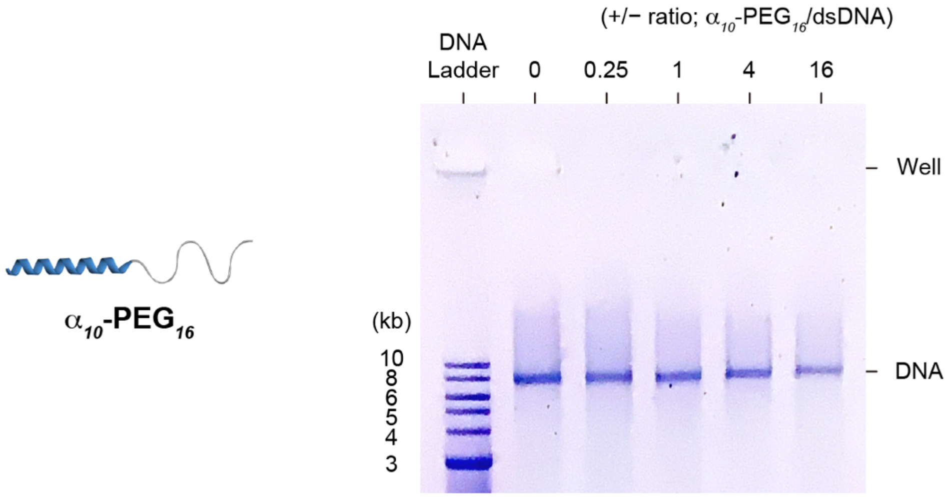


**Supplementary Figure 23.** EMSA of the linear plasmid DNA with $\alpha_{10}$-PEG$_{16}$.

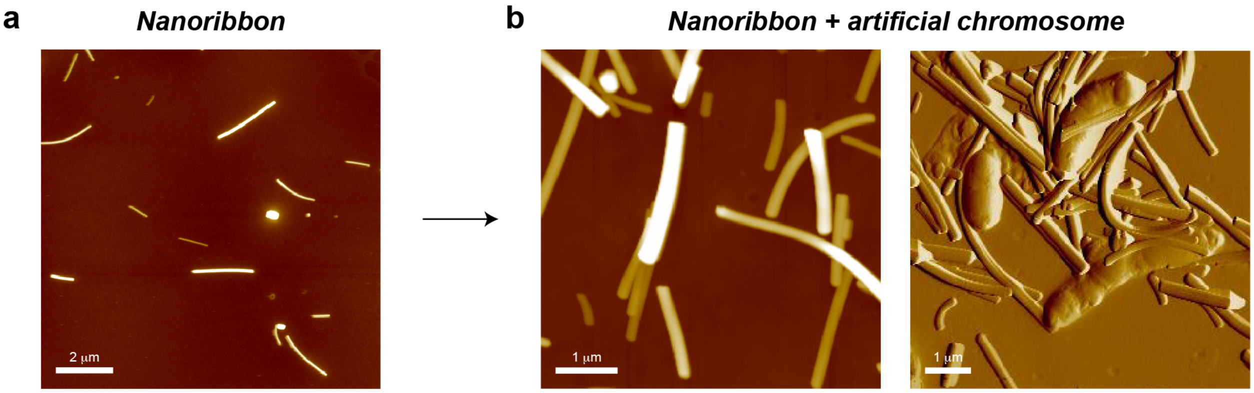


**Supplementary Figure 24.** AFM images of the PD complexes made of R$_4$-$\alpha_{10}$-PEG$_{16}$ and the linear plasmid DNA. The charge ratio (+/–) was 1. **a,** The PD complexes (nanoribbons) incubated for one day. **b,** The PD complexes (nanoribbons + artificial chromosomes) incubated for 3 months.

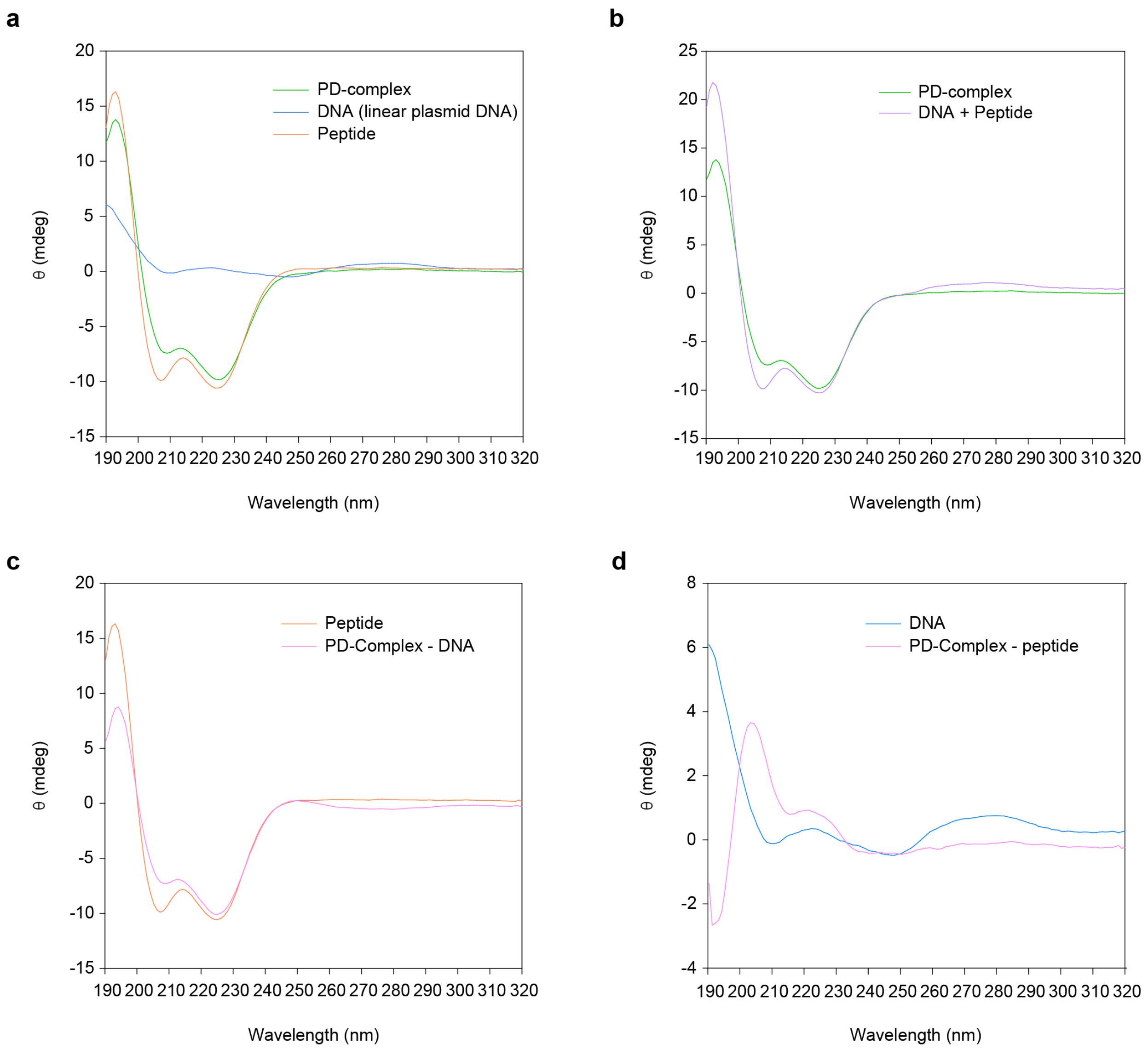


**Supplementary Figure 25. a–d,** CD spectra of the peptide/DNA complexes (PD complexes) and appropriate difference spectra. Peptide = $R_1$-$\alpha_{10}$-$PEG_{16}$; DNA = the linear plasmid DNA.

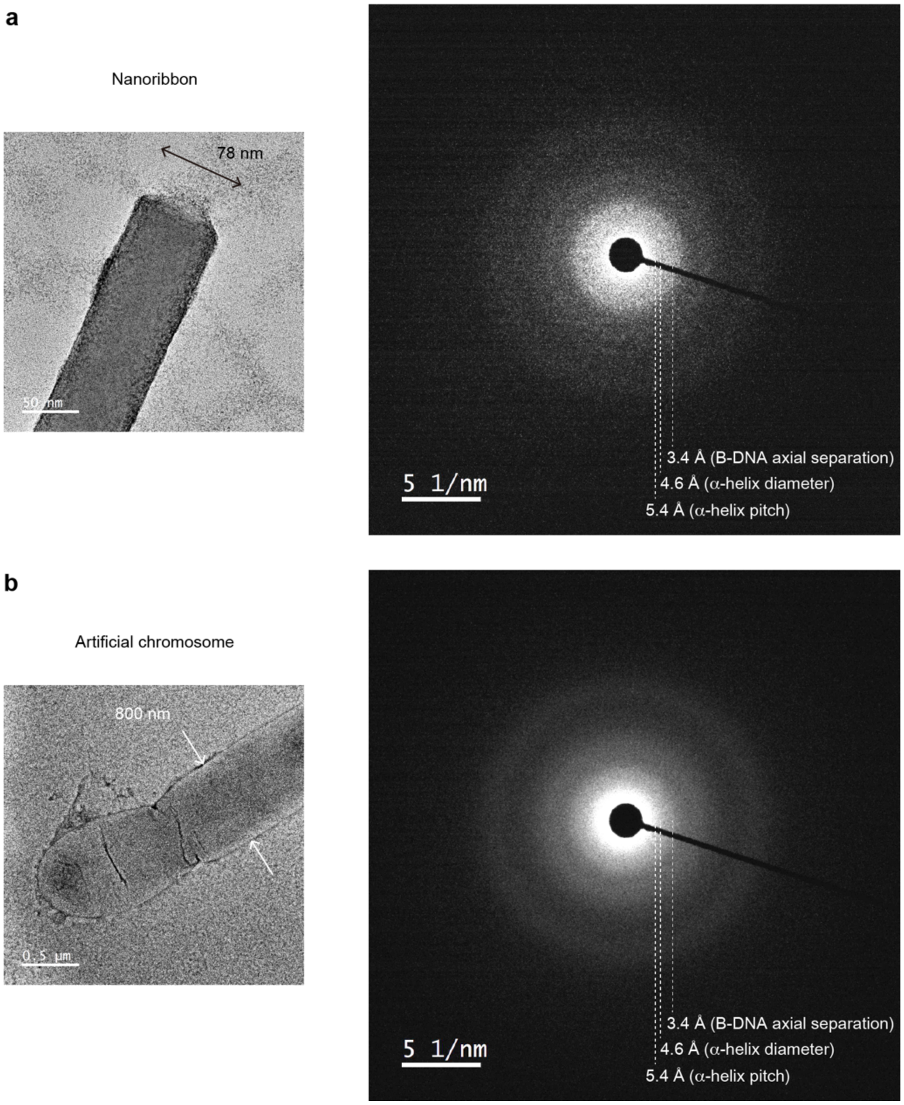


**Supplementary Figure 26.** Selected area electron diffraction (SAED) pattern. **a,** The nanoribbon. **b,** Artificial chromosome.

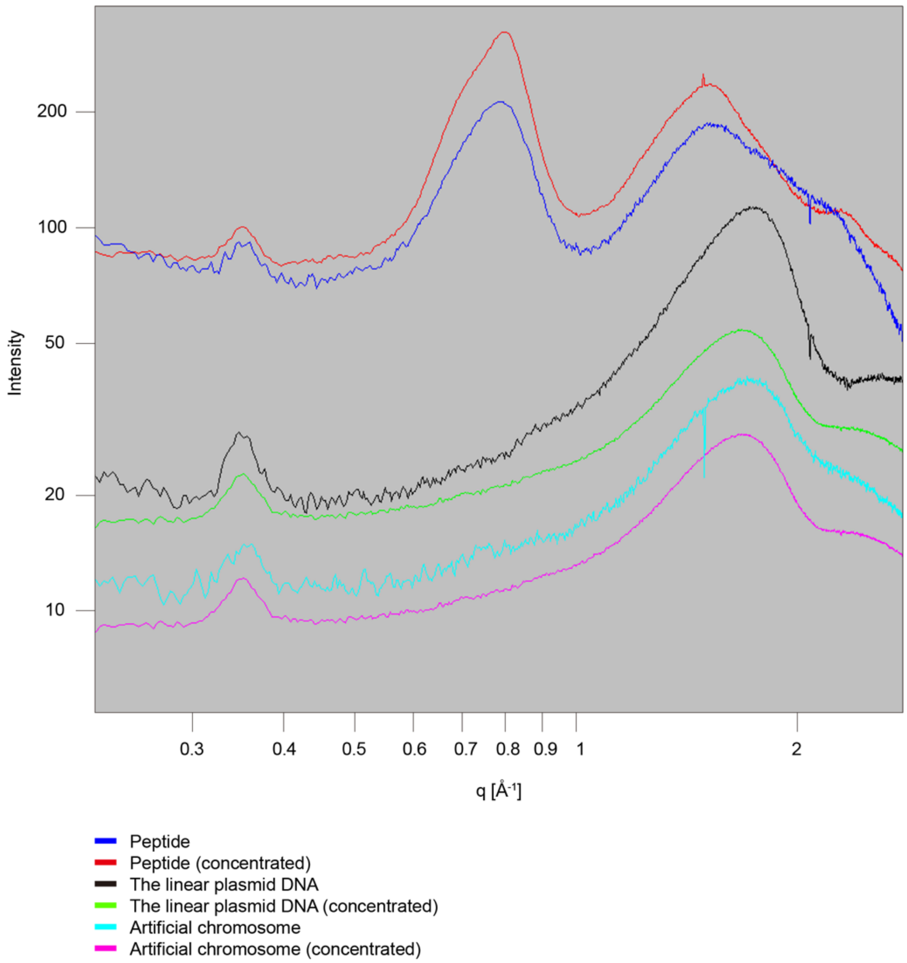


**Supplementary Figure 27.** Synchrotron wide-angle X-ray scattering (WAXS) analyses in solution. Peptide = $R_{1}$-$\alpha_{10}$-$PEG_{16}$; DNA = the linear plasmid DNA.

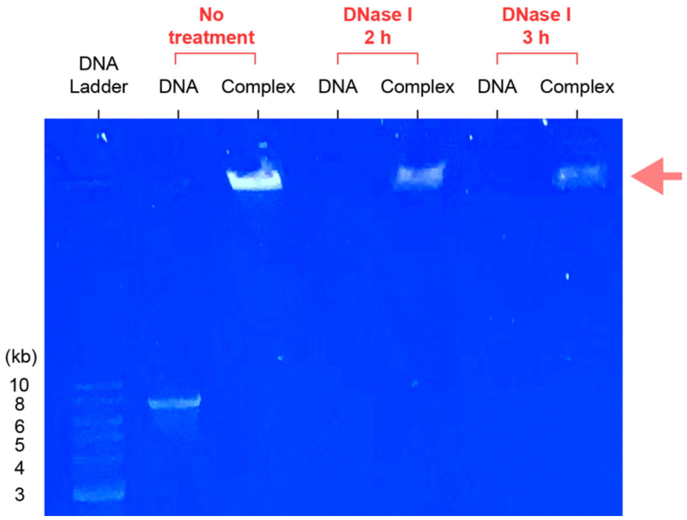


**Supplementary Figure 28.** Time-dependent DNase protection assay for the PD-complex.

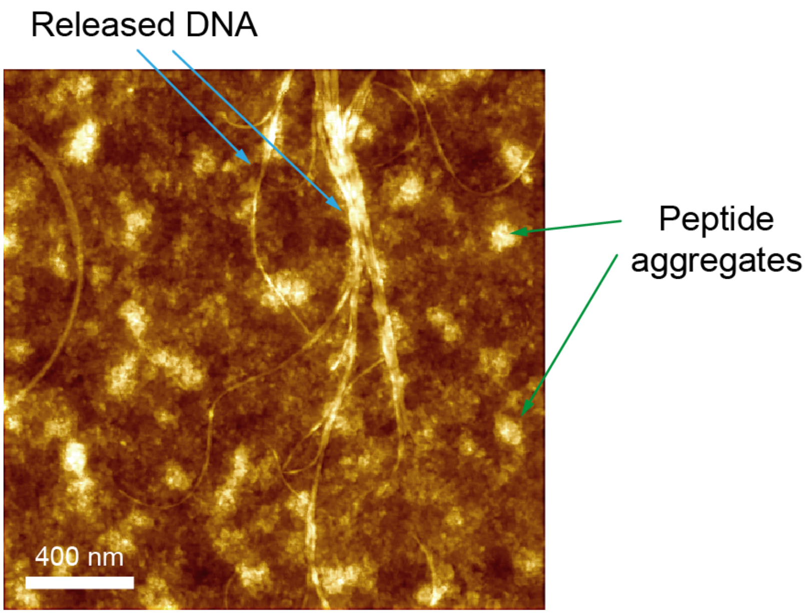


**Supplementary Figure 29.** Release of DNA upon the exposure of the PD-complexes to a static magnetic field (0.1 T) for 2 weeks.

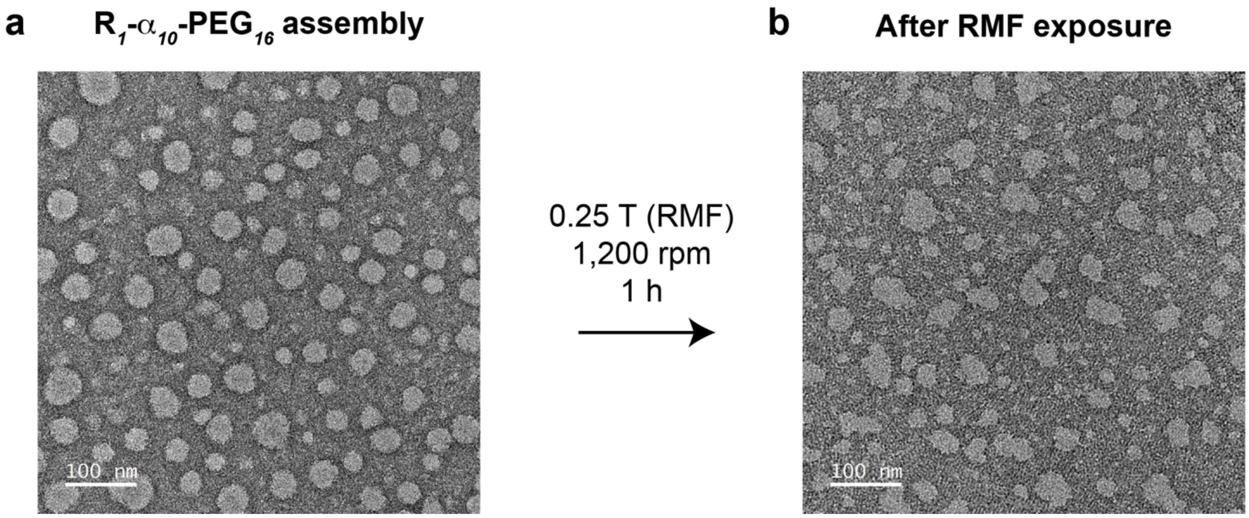


**Supplementary Figure 30. a**,**b**, Effect of RMF exposure on the self-assembled nanostructures from $R_{1}$-$\alpha_{10}$-$PEG_{16}$.

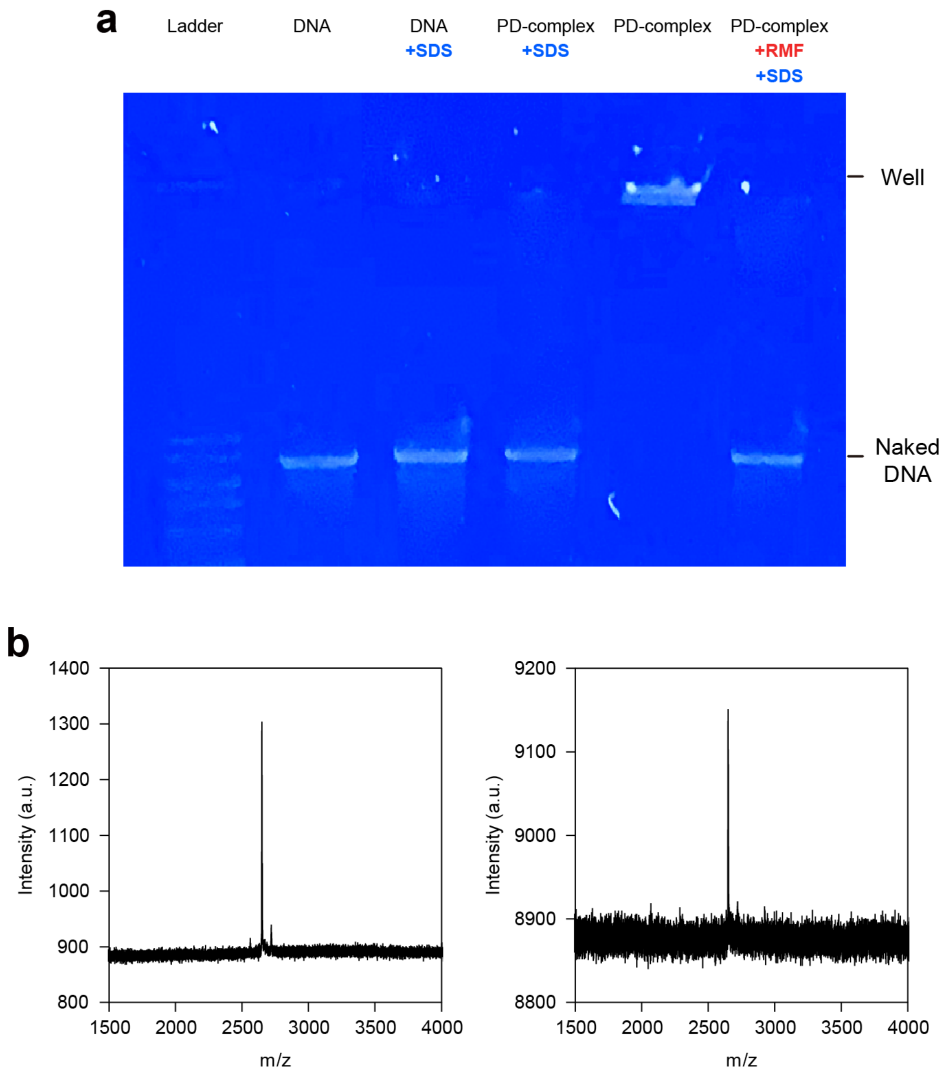


**Supplementary Figure 31.** Intactness of the linear plasmid DNA and the peptide ($R_1$-$\alpha_{10}$-$PEG_{16}$) after incubation. **a,** The integrity of the DNA after incubation. Lane 3: SDS was used to release the DNA from the PD-complex. Lanes 4 and 5: PD-complexes were incubated for 2 weeks. **b,** MALDI-TOF MS spectra. Intactness of the peptide after 2 weeks of incubation (left) and 1 h of RMF treatment (right).

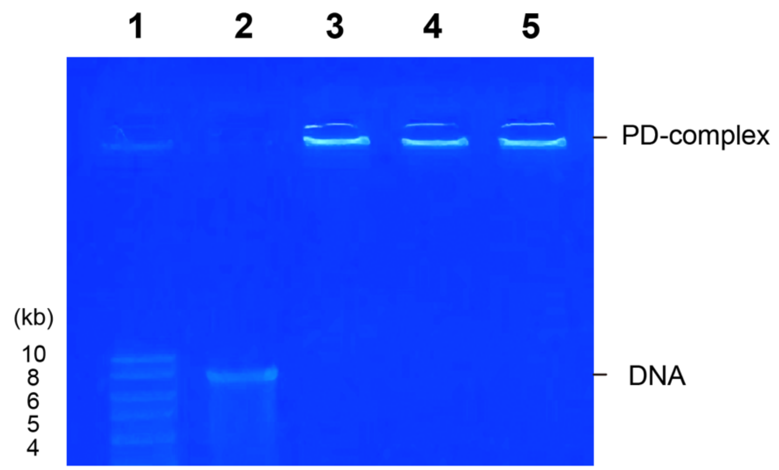


**Supplementary Figure 32.** Influence of MF on the complex formation between the peptide ($R_1$-$\alpha_{10}$-$PEG_{16}$) and the linear plasmid DNA. Lane 1: DNA ladder, lane 2: the plasmid DNA, lane 3: Mixture of the peptide and the DNA, lane 4: Mixture of the peptide and the DNA in the presence of static MF (0.25 T, 1 h), lane 5: Mixture of the peptide and the DNA in the presence of RMF (0.25 T, 1,200 rpm, 1 h)

## References

1. Choi, S. J. *et al.* Differential self-assembly behaviors of cyclic and linear peptides. *Biomacromolecules* **13**, 1991-1995 (2012).
